# Rhaponitin Reverses Cisplatin Resistance and Impairs Cancer Stemness Through HIF‐1α/MCT4/Wnt Pathway in Tongue Squamous Cell Carcinoma

**DOI:** 10.1002/kjm2.70069

**Published:** 2025-07-03

**Authors:** Yuan Wu, Xiao‐Wen Wan, Lin Jiang, Wei Wang, Jia‐Jun Zhu, Yi‐Sen Shao

**Affiliations:** ^1^ Department of Oral and Maxillofacial Surgery Affiliated Hospital of Jiangxi University of Traditional Chinese Medicine Nanchang Jiangxi China

**Keywords:** cisplatin, hypoxia‐inducible factor, rhaponitin, tongue carcinoma

## Abstract

Rhaponitin (Rha) possesses anti‐tumor activity and mediates the transcriptional activity of hypoxia‐inducible factor (HIF)‐1α that affects cisplatin (Cis) resistance. However, whether Rha can lessen Cis resistance in tongue squamous cell carcinoma (TSCC) by mediating HIF‐1α activity is unclear. Cis‐resistant SCC9 (SCC9‐CisR) cells were treated with Cis, Rha, or Cis plus Rha to explore the effect of Rha on Cis resistance using a cell counting kit‐8, flow cytometry, and tumor sphere formation assays. Stemness markers CD44 and SOX2 and HIF‐1α mRNA levels were detected by quantitative PCR. The GSE115119 database and plugin iRegulon were employed to select target genes mediated by HIF‐1α. Protein levels of HIF‐1α, monocarboxylate transporter 4 (MCT4), and the Wnt/β‐catenin pathway were measured by western blot. Subcutaneous xenograft models were constructed to explore the efficacy of Rha in combating Cis resistance. Rha repressed the growth and stemness of SCC9‐CisR cells in vitro and in vivo. HIF‐1α protein levels were markedly elevated in SCC9‐CisR cells, yet Rha treatment attenuated the transcriptional activity of HIF‐1α but not HIF‐1α mRNA levels. Rha plus Cis repressed the viability and stemness of SCC9‐CisR cells, but not HIF‐1α‐knockdown SCC9‐CisR cells, compared with Cis alone. Rha‐induced stemness inhibition and apoptosis in SCC9‐CisR cells were overridden after HIF‐1α overexpression. Rha inhibited the Wnt/β‐catenin signaling by regulating the HIF‐1α/MCT4 axis. In conclusion, Rha reduced cell stemness and enhanced Cis sensitivity in TSCC, which was achieved possibly via suppressing the Wnt/β‐catenin signaling through mediation of the HIF‐1α/MCT4 axis.

## 1 Introduction

Tongue squamous cell carcinoma (TSCC) is the most common type of oral cancer and has a high degree of malignancy, strong metastatic and invasive capabilities, and a poor prognosis [1]. The main treatment strategies for TSCC include surgery combined with radiotherapy, chemotherapy, targeted drug therapy, or immunotherapy [2]. However, local infiltration or lymph node metastasis occurs in most patients with TSCC at the time of diagnosis, resulting in a poor response to treatment [3].

Cisplatin (Cis) is a first‐line chemotherapeutic agent used for the treatment of solid tumors, including TSCC [4]. Cis exerts antitumor effects by interfering with DNA synthesis and repair in cancer cells [5]. However, the application of Cis is limited owing to its side effects (e.g., ototoxicity, peripheral neuropathy, and nephrotoxicity) and drug resistance after long‐term administration [5]. Hence, developing a strategy for TSCC treatment using low‐dose Cis in combination with other drugs is essential to minimize the side effects and chemoresistance associated with Cis.

Hypoxia is a hallmark of solid tumors, including TSCC. A body of evidence has identified that hypoxia is a major factor in TSCC progression [6]. Hypoxia activates the hypoxia‐inducible factor (HIF), which mediates subsequent molecular responses. The activity of prolyl hydroxylase is diminished under hypoxic conditions, thereby allowing HIF‐1α to accumulate in the nucleus and form a complex with HIF‐1β [7]. The complex binds to hypoxia‐responsive elements (HREs) in the promoter regions of target genes, such as p53 and vascular endothelial growth factor (VEGF), enabling transcriptional activation [8, 9]. Transcription of these target genes promotes physiological changes associated with chemotherapy resistance, including the activation of drug efflux and inhibition of senescence, ferroptosis, and apoptosis [10]. Studies have shown that HIF‐1α overexpression is significantly associated with poor overall and disease‐free survival in patients with TSCC [11]. Moreover, HIF‐1α overexpression facilitates the growth and invasion of TSCC cells [12]. A recent study showed that HIF‐1α‐mediated METTL3 transcription increases Cis resistance in oral carcinoma [13]. Thus, targeting HIF‐1α is an attractive strategy to mitigate Cis resistance in patients with TSCC.

Traditional Chinese medicines (TCMs) are important anticancer agents because of their low toxicity, high efficacy, and potential to reverse multidrug resistance [14]. Rhaponitin (Rha), isolated from the root of *Rheum undulatum* L., has various beneficial properties such as anti‐fibrotic, anti‐diabetic, anti‐inflammatory, and anti‐tumor properties [15]. It decreases the metastatic and angiogenic capacities of MDA‐MB231 cells by inhibiting the HIF‐1α pathway [16]. Our previous study demonstrated that Rha represses the migration and invasion of TSCC cells in vitro via suppression of HIF‐1α activity [17]; however, whether Rha can lessen Cis resistance in TSCC by mediating HIF‐1α‐related pathways is unclear.

In this study, we investigated the effect of Rha on Cis resistance in Cis‐resistant SCC9 (SCC9‐CisR) cells and subcutaneous xenograft models. In addition, whether Rha can attenuate Cis resistance by mediating the HIF‐1α‐associated pathway was explored. This study provides evidence for the therapeutic application of Rha against Cis resistance in TSCC.

## 2 Materials and Methods

### 2.1 Cell Culture

The TSCC cell line SCC9 (#CL‐0571; Procell, Wuhan, China) was validated by STR analysis and cultured in DMEM/F12 (#PM150312; Procell) plus 10% FBS (#10100 Thermo, Waltham, MA, USA), 400 ng/mL hydrocortisone (#50‐23‐7; Invivochem, Guangzhou, China), and 1% penicillin–streptomycin (#PB180120; Procell) at 37°C in a humidified 5% CO_2_ atmosphere.

SCC9‐CisR cells were constructed as previously described [18]. To establish stable SCC9‐CisR cells, we treated SCC9 cells with Cis (#P4394; Sigma, St. Louis, MO, USA) at gradually increasing concentrations from 10^−7^ mol/L to 10^−5^ mol/L.

### 2.2 Cell Transfection

Two small interfering RNAs directed against HIF‐1α (siHIF‐1α#1: 5′‐TGGTTACTCAGCACTTTTAGATG‐3′ and siHIF‐1α#2: 5′‐CACTTTTAGATGCTGTTTATAAT‐3′) and the negative control siNC were synthesized by Sangon (Shanghai, China). The pCMV6‐HIF‐1α vector (HIF‐1α oe; #SR300295; OriGene, MD, USA) was used for HIF‐1α overexpression in SCC9 and SCC9‐CisR cells, with the empty vector as control. Transfection was performed using the RiboFECT CP Transfection Kit (Ribobio, Guangzhou, China) according to the manufacturer's instructions.

### 2.3 Drug Treatments

Cis and Rha (> 99%; #155–58‐8; Abmole, Shanghai, China) were dissolved in saline and dimethyl sulfoxide (DMSO) and diluted in the culture medium. SCC9‐CisR cells were processed with different concentrations of Rha (0, 5, 10, 20, 40, and 60 μM) to assess the cytotoxicity of Rha. SCC9 and SCC9‐CisR cells were treated with different concentrations of Cis (0, 2, 4, 6, 8, 10, and 12 μM) for analysis of the half‐maximal inhibitory concentration (IC_50_) of Cis on these two types of cells. For Cis treatment alone, SCC9 and/or SCC9‐CisR cells were subjected to Cis treatment (4.140 μM = the IC_50_ of Cis on SCC9 cells) for 24 h, with an equal amount of saline as a control. For Rha treatment alone, SCC9‐CisR cells were treated with Rha (10 μM) for 24 h, with an equal amount of DMSO as a control. For the combination of Cis and Rha, SCC9‐CisR cells were treated with or without 10 μM Rha for 12 h, followed by exposure to 4.14 μM Cis for 24 h.

### 2.4 Cell Counting Kit‐8 (CCK‐8) Assay

SCC9 and/or SCC9‐CisR cells were seeded into 96‐well plates (1 × 10^3^ cells/well) and treated with indicated drugs for 24 h. Subsequently, the CCK‐8 reagent (10 μL; #C0037; Beyotime, Shanghai, China) was added. Two hours later, the optical density was measured at 450 nm using a microplate reader (Infinite 200 PRO, TECAN, Männedorf, Switzerland). IC_50_ values were determined using inhibition curves.

### 2.5 Flow Cytometry Analysis of Cell Apoptosis

For apoptosis analysis, differentially treated SCC9 and/or SCC9‐CisR cells were harvested and stained using an Annexin V‐FITC/propidium iodide double staining kit (#CA1020; Solarbio, Beijing, China) according to the manufacturer's instructions. Double‐stained SCC9 and/or SCC9‐CisR cells underwent flow cytometry (BD Biosciences, San Jose, CA, USA) analysis. Apoptosis analysis was performed using the FlowJo v10 software (FlowJo LLC, Ashland, OR, USA).

### 2.6 Tumor Sphere Formation Assay

SCC9 and/or SCC9‐CisR cells (1 × 10^3^) were seeded in non‐adherent 6‐well plates. The spheroid culture medium comprised serum‐free DMEM/F12 medium, supplemented with B27 (20 ng/mL; #17504044; Thermo), human EGF (20 ng/mL; #92701ES60; Yeasen, Shanghai, China), and bFGF (20 ng/mL; #91334ES50; Yeasen). After 14 days of incubation, the number of spheroids was recorded under a microscope (Olympus, Tokyo, Japan), and images were captured. The number of spheres (> 50 μm) was analyzed using ImageJ software (NIH, Bethesda, MD, USA).

### 2.7 Reverse Transcription‐Quantitative PCR (RT‐qPCR) Analysis

Total RNA of cultured cells or subcutaneous xenograft tumors was extracted using MolPure Cell/Tissue Total RNA Kit (#19221ES; Yeasen, Shanghai, China). The purity of the extracted total RNA was measured using a UV spectrophotometer (Thermo), and samples that met the requirements showed A260/A280 ranging from 1.8 to 2. A total of 1 μg RNA was reverse‐transcribed into cDNA using the Hifair AdvanceFast 1st Strand cDNA Synthesis Kit (#11149ES; Yeasen). Quantitative PCR was conducted using an ABI7500 Real Time PCR system (Thermo) with the Hieff UNICON Universal Blue qPCR SYBR Green Master Mix (#11184ES; Yeasen). Primer sequences were synthesized by Sangon (listed in Table S1). Relative expression levels were calculated using the 2^−ΔΔCq^ method [19].

### 2.8 Western Blot

Total protein from cultured cells or subcutaneous xenograft tumors was extracted using a radioimmunoprecipitation assay lysis buffer (#P0013B; Beyotime). After the protein concentration was analyzed using a BCA protein concentration assay kit (#P0010; Beyotime), the protein samples were subjected to heat treatment and sodium dodecyl sulfate‐polyacrylamide gel electrophoresis, and transferred onto a polyvinylidene fluoride membrane. After blocking with QuickBlock buffer (#P0228; Beyotime), the membranes were incubated with primary antibodies overnight at 4°C, followed by incubation with the appropriate secondary antibody for 2 h. Detailed antibody information is presented in Table S2. Protein bands were detected using an enhanced chemiluminescence detection system (#P0018; Beyotime), according to the manufacturer's instructions.

### 2.9 Dual‐Luciferase Reporter Assay

SCC9‐CisR cells (1 × 10^3^) were seeded into 96‐well plates and allowed to attach for 24 h, followed by co‐transfection of the pRL‐SV40 plasmid with the pGL3‐HRE luciferase plasmid containing five copies of HREs from the human VEGF genes. After transfection, the cells were treated with or without Rha (0, 5, 10, 20, 40, and 60 μM) for 24 h. Luciferase assays were performed using the Dual‐Glo Luciferase Assay System (Promega, Madison, WI, USA) per the manufacturer's directions.

### 2.10 Animal Experiments

Male BALB/c nude mice (4‐6‐week‐old, weighing 18–23 g; Animal Experimental Science Center of Nanchang University, Nanchang, China) were maintained under a condition system with temperature (24°C ± 1°C), humidity (50%–60%), and lighting (a 12:12 h light/dark cycle). Housing and experimental procedures followed the National Institutes of Health Guidelines for the Care and Utilization of Laboratory Animals. This study was approved by the Animal Ethics Committee of the Affiliated Hospital of the Jiangxi University of Traditional Chinese Medicine.

To prepare a subcutaneous tumor model, we subcutaneously injected SCC9 or SCC9‐CisR cells (5 × 10^5^ cells) suspended in PBS (100 μL) into the dorsal flanks of BALB/c mice. Two weeks later, the BALB/c mice injected with SCC9 cells were administered saline or Cis (4 mg/kg) via gavage. BALB/c mice injected with SCC9‐CisR cells were randomly divided into four groups (*n* = 6): (1) SCC9‐CisR (saline + DMSO; gavage, daily); (2) SCC9‐CisR + Cis [Cis (4 mg/kg) + DMSO; gavage, daily]; (3) SCC9‐CisR + Rha [saline + Rha (20 mg/kg); gavage, daily]; and (4) SCC9‐CisR + Cis + Rha [Cis (4 mg/kg) pus Rha (20 mg/kg); gavage, daily] (*n* = 6). The drug was administered daily for 30 days. Changes in tumor volume were measured every 4 days using a vernier caliper and were calculated as follows: volume (mm^3^) = length × width^2^ × 0.5. After the mice were sacrificed, tumor xenografts were harvested for subsequent analysis.

### 2.11 Bioinformatics Analysis

RNA‐seq expression data associated with SCC9 and SCC9‐CisR cells were downloaded from the GEO database (GSE115119) for further analysis of differentially expressed genes (DEGs; *p* < 0.05, |fold change| ≥ 1). A plugin iRegulon in Cytoscape was used to select transcription factors (TFs) mediated by HIF‐1α. DEGs and predicted TFs were analyzed using Venn analysis to screen for TFs that may be associated with the development of Cis resistance in TSCC.

### 2.12 Statistical Analysis

All data were analyzed using GraphPad Prism Software (Ver. 8.0.2; GraphPad, La Jolla, CA, USA) and expressed as the mean ± standard error of means (SEM). The two groups were compared using a two‐sided unpaired *t*‐test. Multiple comparisons were performed using one‐ or two‐way analysis of variance (ANOVA). Statistical significance was set at *p* < 0.05.

## 3 Results

### 3.1 Establishment of the SCC9‐CisR Cell Line

**FIGURE 1:**
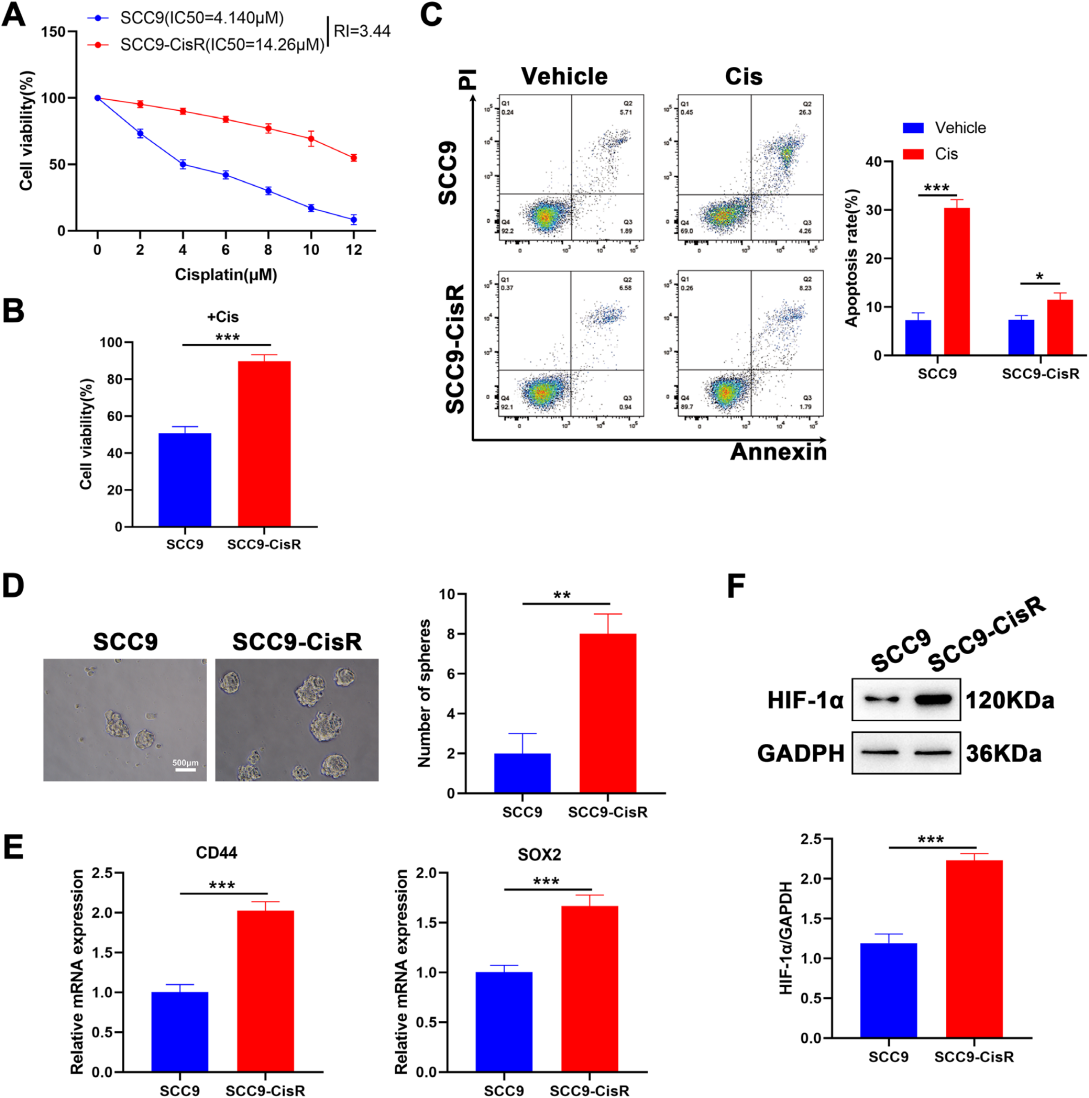
Establishment and assessment of SCC9‐CisR cells. (A) CCK‐8 analysis of the viability of SCC9 and SCC9‐CisR cells after 24 h of treatment across Cis concentrations (*n* = 3). (B) CCK‐8 assessment of the viability of SCC9 and SCC9‐CisR cells after 24 h of treatment with a specified Cis concentration (4.140 μM) (*n* = 3). (C) Flow cytometry detection of the apoptosis of SCC9 and SCC9‐CisR cells after 24 h of treatment with vehicle (DMSO) or Cis (4.140 μM) (*n* = 3). (D) Tumor sphere formation assays were performed to detect the stemness of SCC9 and SCC9‐CisR cells after 14 days of treatment with Cis (4.140 μM) (*n* = 3). Scale bar: 500 μm. (E) Relative mRNA levels of CD44 and SOX2 in SCC9 and SCC9‐CisR cells were detected by RT‐qPCR (*n* = 3). (F) Western blot analysis of HIF‐1α protein levels in SCC9 and SCC9‐CisR cells (*n* = 3). Data are presented as mean ± SEM. *p < 0.05, **p < 0.01, and ***p < 0.001, two‐way ANOVA followed by Tukey's post hoc test (A, C); two‐tailed unpaired *t*‐test (B, D–F).

To elucidate the effect of Rha on Cis resistance in TSCC, a Cis‐resistant TSCC cell line, SCC9‐CisR, was established by continuous exposure to increasing concentrations of Cis for 24 h. CCK‐8 assays showed an apparent elevation in the IC_50_ value of SCC9‐CisR cells (14.26 μM) versus its parental cell line (4.140 μM) (Figure 1A). We also analyzed the resistance index (RI), which is the ratio of the IC_50_ of drug‐resistant cells to the IC_50_ of parental cells for the same drug. The RI was 3.44, as seen in Figure 1A. To validate the resistance of SCC9‐CisR cells to Cis, SCC9‐CisR cells and their parental cells were treated with Cis (4.140 μM) for 24 h. SCC9‐CisR cells demonstrated higher viability under Cis treatment than their parental cells (Figure 1B). Apoptosis and stemness characteristics of SCC9‐CisR cells were also evaluated. Flow cytometry assays showed that Cis treatment induced apoptosis in SCC9 and SCC9‐CisR cells; however, apoptosis markedly decreased in SCC9‐CisR cells compared with that in SCC9 cells (Figure 1C). SCC9‐CisR cells exhibited a stronger sphere‐forming ability than their parental cells, as evidenced by enlarged and increased tumor spheroids (Figure 1D). Consistently, SCC9‐CisR cells showed higher mRNA levels of stem cell markers CD44 and SOX2 (Figure 1E). Because HIF‐1α expression is associated closely with resistance to radiotherapy [20], HIF‐1α protein levels were determined. To mimic hypoxia and induce HIF‐1α stabilization, SCC9 and SCC9‐CisR cells were pretreated with 150 μM cobalt (II) chloride for 12 h before treatment. As expected, HIF‐1α protein levels were markedly increased in SCC9‐CisR cells compared with those in SCC9 cells treated with Cis (Figure 1F). We then established HIF‐1α‐knockdown SCC9‐CisR cells to validate the role of HIF‐1α. As illustrated in Figure S1A,B, HIF‐1α mRNA and protein levels were lowly expressed in SCC9‐CisR cells after siHIF‐1α#1 or siHIF‐1α#2 transfection, and siHIF‐1α#2 was used for subsequent analyses. Moreover, HIF‐1α‐knockdown SCC9‐CisR cells showed a lower IC_50_ value for Cis than the siNC group (Figure S1C). In addition, HIF‐1α‐overexpressed SCC9 cells were constructed, and the transcriptional and translational levels of HIF‐1α were promoted after the introduction of exogenous HIF‐1α (Figure S1D,E). We also observed an elevated IC_50_ value for Cis in HIF‐1α‐overexpressed SCC9 (Figure S1F). Collectively, these data highlight a successful construction for SCC9‐CisR cells, and the resistance of SCC9‐CisR to Cis is associated with HIF‐1α upregulation.

### 3.2 Rha Decreases Cis Resistance and Represses Cell Stemness in SCC9‐CisR Cells

**FIGURE 2:**
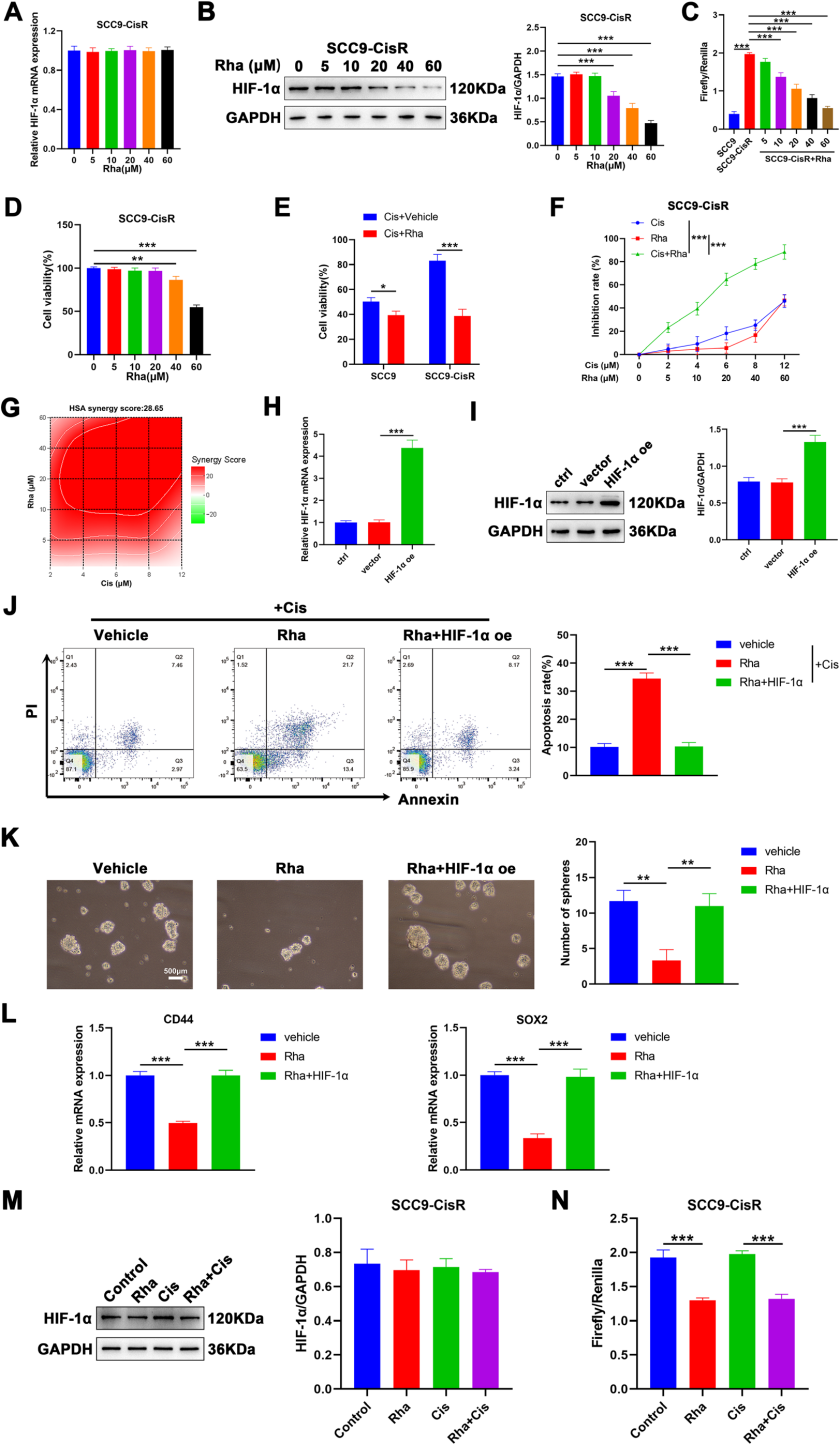
Rha may reduce Cis resistance in SCC9‐CisR cells by repressing the transcriptional activity of HIF‐1α. (A, B) RT‐qPCR and western blot analyses of HIF‐1α mRNA levels in SCC9‐CisR cells after 24 h of treatment with different concentrations of Rha (0, 5, 10, 20, 40, 60 μM) alone (*n* = 3). (C) Dual‐luciferase reporter assays were executed to assess the effect of Rha alone on the transcriptional activity of HIF‐1α in SCC9‐CisR cells (*n* = 3). (D) CCK‐8 assay evaluating the effect of Rha alone on the viability of SCC9‐CisR cells (*n* = 3). (E) Viability of SCC9 and SCC9‐CisR cells after 24 h of treatment with Cis (4.140 μM) + vehicle (DMSO) or Cis (4.140 μM) + Rha (10 μM) (*n* = 3). (F) The inhibitory effect of Cis plus Rha on the viability of SCC9‐CisR cells compared with that of Cis or Rha alone was determined by CCK‐8 assays (*n* = 3). (G) The calculation of synergy scores was performed using SynergyFinder. (H, I) After transfection with vector or HIF‐1α oe, HIF‐1α mRNA and protein levels in SCC9‐CisR cells were evaluated by RT‐qPCR and western blot (*n* = 3). (J–L) SCC9‐CisR cells were processed as follows: Vehicle, Rha, or Rha + HIF‐1α. The apoptosis of SCC9‐CisR cells was analyzed by flow cytometry (J); the tumor sphere‐forming ability of SCC9‐CisR cells was determined by tumor sphere formation assays (K); relative mRNA levels of CD44 and SOX2 in SCC9‐CisR cells were detected by RT‐qPCR (L) (*n* = 3). (M) HIF‐1α protein levels in SCC9‐CisR cells treated with Rha (10 μM), Cis (4.140 μM), or Rha (10 μM) plus Cis (4.140 μM) were detected by western blot (*n* = 3). (N) The effect of Rha, Cis, or Rha plus Cis on the transcriptional activity of HIF‐1α in SCC9‐CisR cells was determined by dual‐luciferase reporter assay (*n* = 3). Data are presented as mean ± SEM. *p < 0.05, **p < 0.01, and ***p < 0.05, one‐way ANOVA followed by Tukey's post hoc test (A–, H–N); two‐way ANOVA followed by Tukey's post hoc test (E, F).

To explore whether Rha can regulate Cis resistance in SCC9‐CisR cells via mediating HIF‐1α, we detected HIF‐1α mRNA levels in SCC9‐CisR cells treated with different concentrations of Rha. The results showed that Rha treatment did not significantly alter HIF‐1α mRNA levels in SCC9‐CisR cells (Figure 2A). Western blotting showed that Rha treatment significantly repressed HIF‐1α protein levels in a dose‐dependent manner (Figure 2B). To determine whether Rha influences HIF‐1α‐mediated transcriptional activity, we co‐transfected SCC9 and SCC9‐CisR cells with pGL3‐HRE and pRL‐SV40 plasmids, followed by treatment with or without Rha. Luciferase reporter assays indicated that the transcriptional activity of HIF‐1α was elevated significantly in SCC9‐CisR cells compared with that in SCC9 cells. However, Rha treatment significantly attenuated the transcriptional activity of HIF‐1α in SCC9‐CisR cells in a concentration‐dependent manner (10–60 μM) (Figure 2C). Rha treatment suppressed remarkably the viability of SCC9‐CisR cells when its concentration was ≥ 40 μM (Figure 2D). Subsequently, we analyzed the effect of Rha (10 μM, the lowest Rha concentration that inhibits HIF‐1α transcriptional activity) combined with Cis on the viability and apoptosis of SCC9‐CisR cells. The results demonstrated that Rha combined with Cis repressed cell viability in SCC9 and SCC9‐CisR cells, particularly in SCC9‐CisR cells (Figure 2E). Moreover, Rha plus Cis showed a greater inhibitory effect on SCC9‐CisR cells than Cis or Rha alone (Figure 2F), with a synergy score of 28.65 for Rha plus Cis (Figure 2G). Subsequently, we investigated whether HIF‐1α overexpression affects the efficacy of Rha in SCC9‐CisR cells. After HIF‐1α oe transfection, HIF‐1α mRNA and protein levels were upregulated significantly in SCC9‐CisR cells compared with those in the control and vector groups (Figure 2H,I). We observed that Rha promoted the apoptosis of SCC9‐CisR cells and inhibited their tumor sphere‐forming ability, accompanied by reduced CD44 and SOX2 mRNA levels. However, these effects mediated by Rha treatment were reversed following the introduction of ectopic HIF‐1α (Figure 2J–L). Interestingly, while 10 μM Rha did not markedly reduce HIF‐1α protein levels (Figure 2B), it significantly inhibited HIF‐1α transcriptional activity, as measured by the HRE‐luciferase assay (Figure 2C). This suggests that the functional suppression of HIF‐1α may occur at the transcriptional co‐activation or nuclear signaling level rather than through direct protein degradation. Furthermore, cisplatin co‐treatment did not further suppress HIF‐1α protein levels or transcriptional activity (Figure 2M,N), reinforcing the conclusion that Rha is the primary regulatory factor in this pathway. These results collectively indicate that Rha treatment decreases Cis resistance and represses cell stemness in SCC9‐CisR cells, which may be achieved by inhibiting the transcriptional activity of HIF‐1α.

### 3.3 Rha Fails to Mediate Cis Resistance and Cell Stemness in SCC9‐CisR Cells With HIF‐1α Knockdown

**FIGURE 3:**
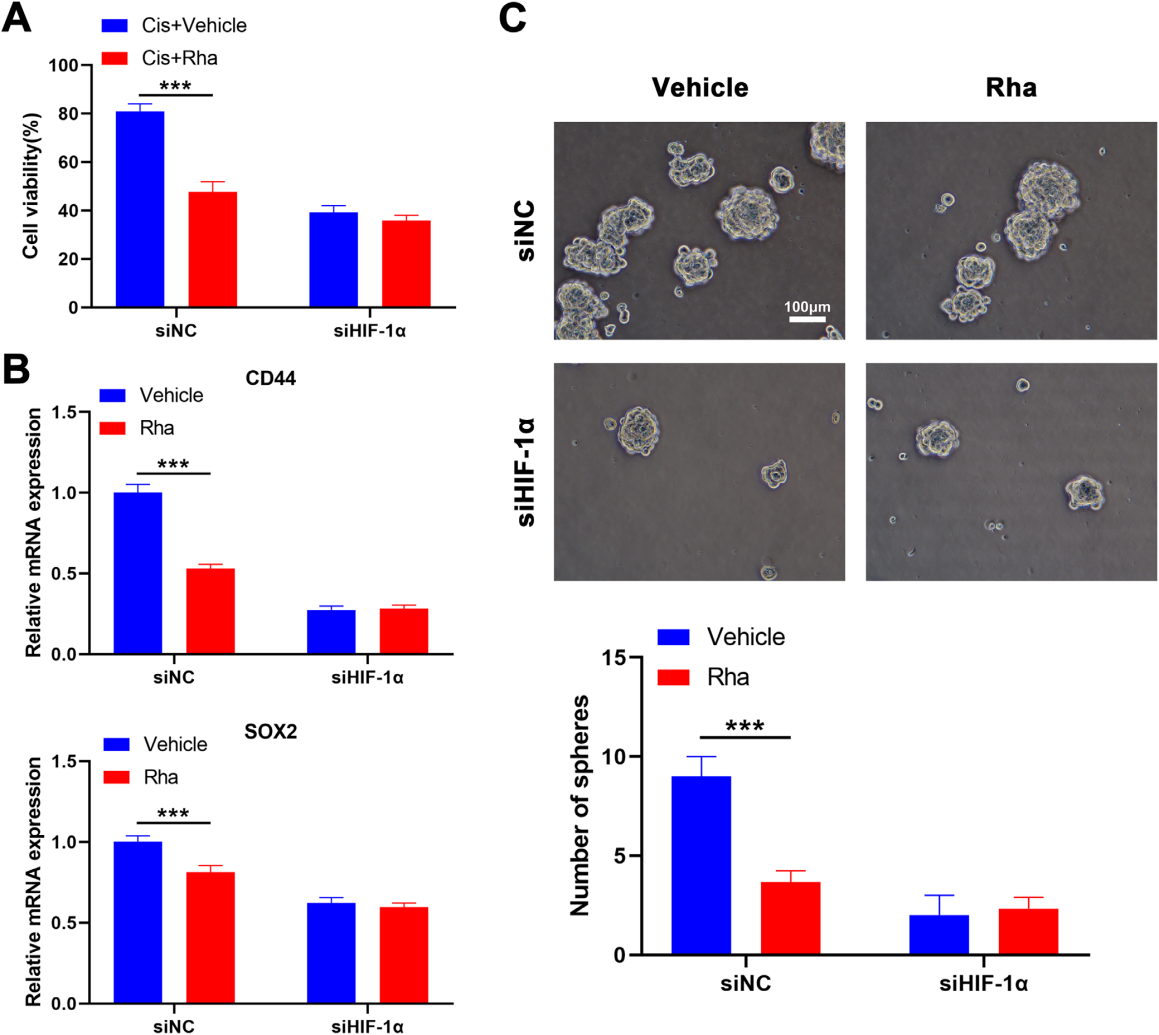
Rha represses cell viability and stemness in SCC9‐CisR cells by mediating HIF‐1α activity. (A) SCC9‐CisR cells were transfected with siNC or siHIF‐1α#2, followed by treatment with Cis (4.140 μM) + vehicle (DMSO) or Cis (4.140 μM) + Rha (10 μM). The viability of SCC9‐CisR cells was determined by CCK‐8 assays (*n* = 3). (B, C) SCC9‐CisR cells were transfected with siNC or siHIF‐1α#2, followed by treatment with vehicle (DMSO) or Rha alone (10 μM). Relative mRNA levels of CD44 and SOX2 in SCC9‐CisR cells were detected by RT‐qPCR (B); the sphere‐forming ability of SCC9‐CisR cells was estimated by tumor sphere‐forming assays (C) (*n* = 3). Scale bar: 100 μm. Data are presented as mean ± SEM. ***p < 0.001, two‐way ANOVA followed by Tukey's post hoc test (A–C).

Next, we further verified whether Rha mediates the stemness of SCC9‐CisR cells through HIF‐1α. Rha combined with Cis repressed the activity of SCC9‐CisR cells, in contrast to Cis alone. However, Rha plus Cis did not significantly affect the activity of SCC9‐CisR cells compared with Cis alone after HIF‐1α knockdown (Figure 3A). As expected, the sphere‐forming ability of SCC9‐CisR cells was reduced following Rha treatment, along with decreased CD44 and SOX2 mRNA levels. However, no significant changes were observed in the sphere‐forming capacity or mRNA levels of CD44 and SOX2 in HIF‐1α‐knockdown SCC9‐CisR cells with or without Rha treatment (Figure 3B,C). These results indicate that Rha mediates cell stemness in SCC9‐CisR cells through HIF‐1α.

### 3.4 Rha Inhibits Cis Resistance in TSCC In Vivo

**FIGURE 4:**
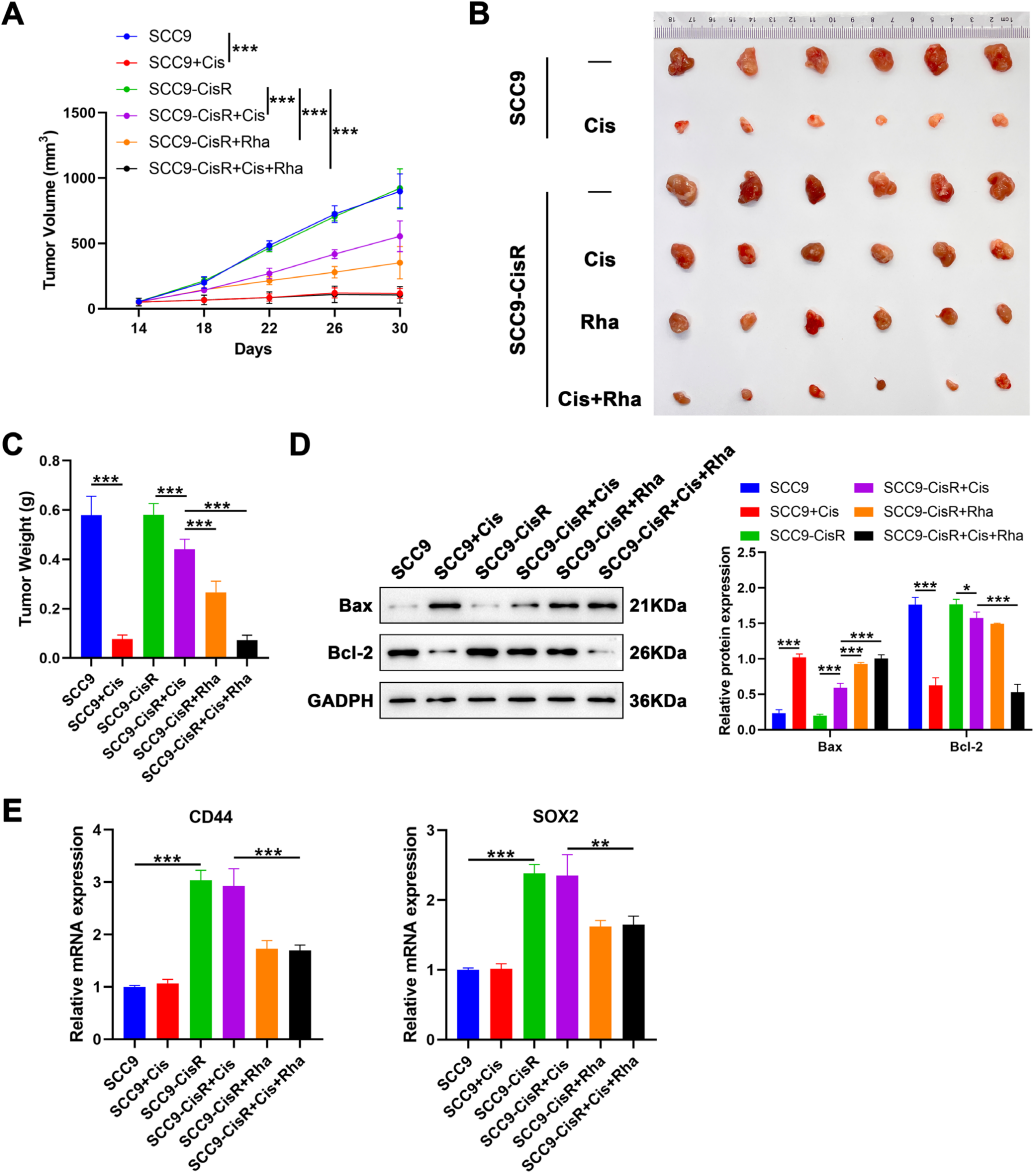
Rha treatment decreases Cis resistance in TSCC in vivo. (A) Changes in tumor volume of mice in different subgroups from days 14 to 30 (*n* = 6). (B) A photograph containing tumor tissues from different groups of mice at the end of the experiment. (C) Average weight of tumors in different groups of mice (*n* = 6). (D) Bax and Bcl‐2 protein levels in different groups of tumor samples were detected by western blot (*n* = 3). (E) CD44 and SOX2 mRNA levels were detected in tumor samples from different groups (*n* = 6). Data are presented as mean ± SEM. ***p < 0.001, one‐way ANOVA followed by Tukey's post hoc test (C, E); two‐way ANOVA followed by Tukey's post hoc test (A, D).

To validate the effect of Rha on Cis resistance in TSCC in vivo, we treated SCC9‐CisR cell‐derived subcutaneous xenografts with Cis, Rha, or Cis plus Rha, with Cis‐treated SCC9 cell‐derived subcutaneous xenografts used as positive controls. The results showed that treatment with Cis, Rha, or Cis plus Rha repressed tumor growth and significantly decreased tumor weight, with the strongest efficacy of Cis plus Rha, followed by Rha and Cis (Figure 4A–C). Moreover, treatment with Cis, Rha, or Cis plus Rha increased Bax protein levels and decreased Bcl‐2 protein levels significantly, but Rha or Cis plus Rha had a stronger action than Cis alone, especially for the Bax protein (Figure 4D). The mRNA levels of CD44 and SOX2 were upregulated in SCC9‐CisR cell‐derived tumors compared with those in tumors derived from SCC9 cells. Rha or Cis plus Rha treatment decreased CD44 and SOX2 mRNA levels in SCC9‐CisR cell‐derived tumors instead of Cis treatment, and Cis combined with Rha worked better than Rha alone (Figure 4E). These results suggest that Rha treatment decreases Cis resistance in TSCC in vivo.

### 3.5 Rha Inhibits the Wnt/β‐Catenin Pathway via Regulating the HIF‐1α/Monocarboxylate Transporter 4 (MCT4) Axis

**FIGURE 5:**
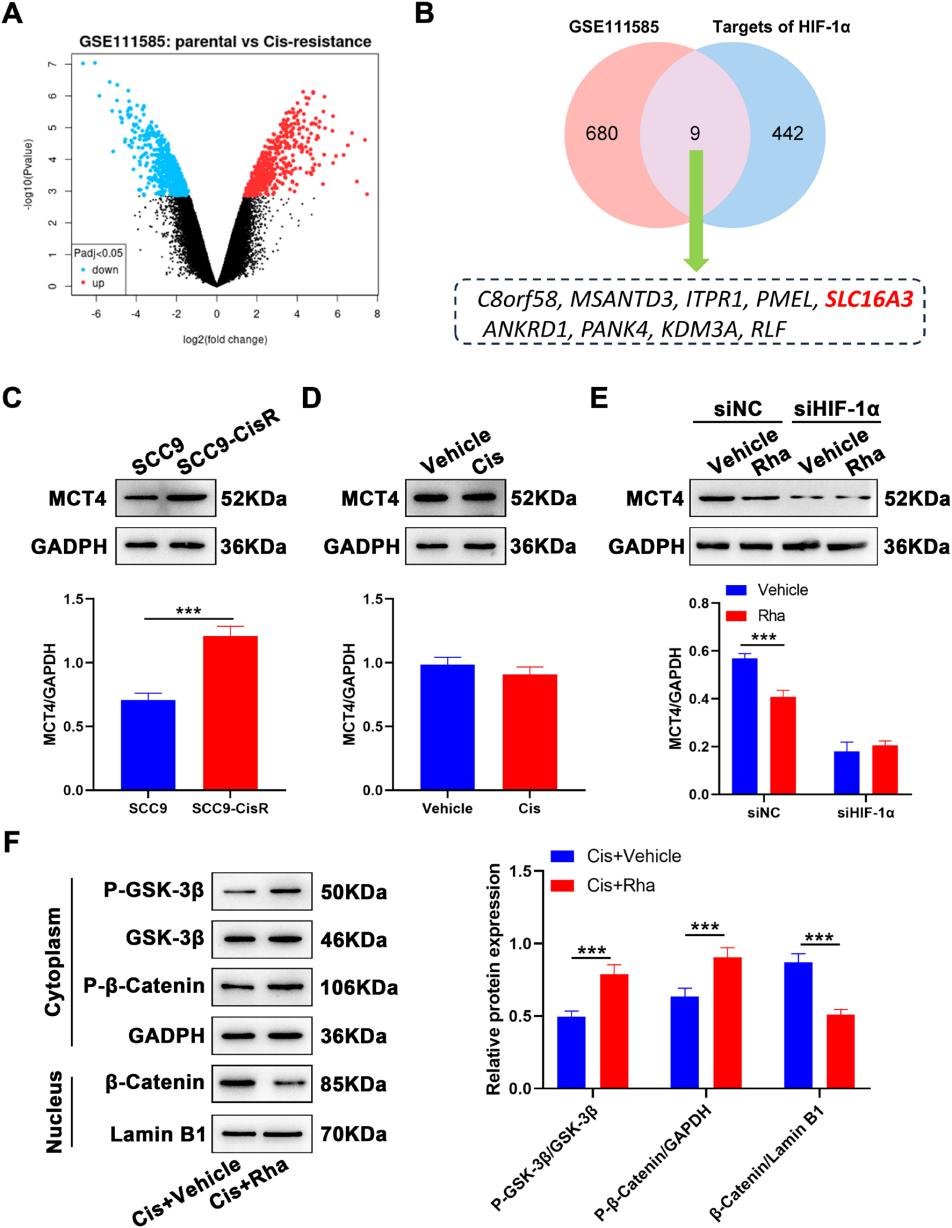
The HIF‐1α/MCT4 axis and the Wnt/β‐catenin signaling are repressed by Rha in SCC9‐CisR cells. (A) Volcano plot of DEGs in GSE111585. (B) Venn diagram of DEGs (GSE111585) and the target of HIF‐1α predicted by the iRegulon plugin. (C) Western blot analysis of MCT4 protein levels in SCC9 and SCC9‐CisR cells without any treatment (*n* = 3). (D) MCT4 protein levels were detected in SCC9‐CisR cells treated with vehicle or Rha alone (*n* = 3). (E) MCT4 protein levels were assessed in siNC/siHIF‐1α‐transfected SCC9‐CisR cells treated with vehicle or Rha alone (*n* = 3). (F) Western blot analysis of P‐GSK‐3β and P‐β‐catenin protein levels in the cytoplasm and β‐catenin protein levels in the nucleus of SCC9‐CisR cells treated with Cis + vehicle or Cis + Rha (*n* = 3). Data are presented as mean ± SEM. **p < 0.01 and ***p < 0.001, two‐tailed unpaired *t*‐test (C, D); two‐way ANOVA followed by Tukey's post hoc test (E, F).

To gain deeper insight into the regulatory mechanism of Rha, we screened the downstream target of HIF‐1α in TSCC. By analyzing the GSE111585 database, we obtained 680 DEGs for SCC9 and SCC9‐CisR cells (*p* < 0.05, |fold change| ≥ 1) (Figure 5A). The obtained DEGs intersected with the target of HIF‐1α predicted using the iRegulon plugin, resulting in a final set of 9 genes: C8orf58, MSANTD3, ITPR1, PMEL, SLC16A3, ANKRD1, PANK4, KDM3A, and RLF (Figure 5B). The SLC16A3 gene encoding the MCT4 protein responsible for the extracellular transport of lactate can be activated by HIF‐1α through binding to HREs [21]. Therefore, we explored whether Rha exerted its effects by mediating MCT4 expression. As expected, MCT4 protein levels were elevated overtly in SCC9‐CisR cells (Figure 5C) but did not change after treatment with Cis (Figure 5D), suggesting that MCT4 is involved in Cis resistance. Additionally, Rha treatment reduced MCT4 protein levels in SCC9‐CisR cells but did not affect MCT4 protein levels in SCC9‐CisR cells following HIF‐1α knockdown, implying that Rha mediates MCT4 protein levels by regulating HIF‐1α transcriptional activity (Figure 5E). The Wnt/β‐catenin signaling pathway located downstream of MCT4 is closely associated with chemotherapy resistance in diverse tumors; thus, we further investigated the effect of Rha combined with Cis on this signaling pathway in SCC9‐CisR cells. The results demonstrated that Rha plus Cis elevated P‐GSK‐3β (Y216) and P‐β‐catenin protein levels in the cytoplasm and decreased β‐catenin protein levels in the nucleus compared with Cis alone, suggesting that Rha elevates GSK‐3β activity and represses nuclear translocation of β‐catenin (Figure 5F). All results indicate that Rha inhibits the Wnt/β‐catenin signaling by mediating the HIF‐1α/MCT4 axis.

## 4 Discussion

Adjuvant radiotherapy combined with chemotherapy in the postoperative period may enhance the control of primary foci and improve the survival rate of patients with advanced‐stage TSCC and those with poor pathological typing [22]. Cis‐based chemotherapy is used for the clinical management of TSCC; however, the resistance to and adverse side effects of Cis impede its efficacy. Hence, the development of drugs with low toxicity and enhanced sensitivity to Cis is essential.

TCM treatment not only relieves the symptoms of patients with cancer but also reduces the side effects and complications caused by chemoradiotherapy [23]. For instance, the ginsenoside Rb1 extracted from *Panax ginseng* reverses Cis resistance in A549/Cis cells by targeting the Hedgehog and ABCB1 pathways [24]. *Rheum undulatum* L. is a TCM, and its roots contain piceatannol, Rha, rhein, and emodin. A previous study reported that Rha plus ceritinib significantly suppresses tumor activity in lung cancer [25]. Hibasami et al. showed that the Rha‐mediated suppressive effect on KATO III cells is achieved by inducing apoptosis [26]. Kim et al. demonstrated that the colony‐forming, migratory, and invasive capacities of MDA‐MB23 cells are reduced following Rha treatment [16]. We previously demonstrated that Rha inhibits migration and invasion of TSCC cells in vitro [17]; however, whether Rha can fight Cis resistance in TSCC is unclear. Our results showed that treatment with Rha plus Cis repressed cell viability compared with Cis or Rha alone. The self‐renewal capacity of tumor stem cells allows them to continuously generate new tumor cells and resist conventional treatments such as chemotherapy and radiotherapy [27]. Rha treatment induced apoptosis and repressed the stemness of SCC9‐CisR cells. Consistent results were also observed in in vivo subcutaneous xenograft experiments, in which treatment with Cis plus Rha repressed tumor stemness, slowed tumor growth, and decreased tumor weight compared with treatment with Cis or Rha alone. These findings suggest that Rha improves Cis resistance in TSCC.

HIF‐1α is a key TF involved in cancer progression and targeted therapy. HIF‐1α has been established to boost TSCC progression by enhancing the transcription of target genes VEGF and adiponectin [8, 12]. Available evidence suggests that HIF‐1α facilitates drug resistance by enhancing the stemness of tumor cells [28]. Notably, Rha has been disclosed to suppress HIF‐1α accumulation and nuclear expression in MDA‐MB23 cells, thus repressing EMT‐associated protein levels [16]. Furthermore, Rha restrains the malignancy of TSCC cells by reducing HIF‐1α activity [17]. Consistent with our previous findings, Rha decreased HIF‐1α protein expression without altering its mRNA levels, suggesting a post‐transcriptional regulatory mechanism. In that study, molecular docking analysis indicated that Rha may bind to the PAS domain of HIF‐1α, which is essential for its dimerization with ARNT and subsequent transcriptional activation. Disruption of this interface could potentially impair HIF‐1α stability and function [29]. In this study, HIF‐1α protein levels were markedly elevated in SCC9‐CisR cells, and HIF‐1α knockdown decreased the resistance of SCC9‐CisR cells to Cis. However, HIF‐1α overexpression elevated Cis resistance for SCC9 cells, indicating that HIF‐1α elevation is associated with Cis resistance in TSCC cells. However, Rha treatment attenuated the transcriptional activity of HIF‐1α but not HIF‐1α mRNA levels in SCC9‐CisR cells. Moreover, Rha (≥ 10 μM) inhibited the transcriptional viability of HIF‐1α in SCC9‐CisR cells but had an inhibitory effect on the viability of SCC9‐CisR cells when its concentration was at ≥ 40 μM. However, Rha or Rha combined with Cis did not significantly affect the viability and stemness of SCC9‐CisR cells after HIF‐1α knockdown compared with their respective controls. In addition, HIF‐1α overexpression reversed Rha‐mediated effects on the apoptosis and stemness of SCC9‐CisR cells. These findings suggest that Rha mediates Cis resistance in SCC9‐CisR cells by modulating the transcriptional activity of HIF‐1α. These data collectively suggest that Rha primarily modulates TSCC cell behavior via inhibition of HIF‐1α, and additional agents such as cisplatin may not further enhance this regulatory axis.

CD44 and SOX2 play a central role in stemness maintenance, metastasis, and drug resistance in TSCC, and their co‐expression may serve as prognostic markers [30, 31]. The biological significance of mRNA expression changes in molecular biology research is not solely a function of the absolute value of the fold change. Information on gene function should be integrated with the outcomes of functional validation. In this study, the stemness markers CD44 and SOX2 were altered approximately 2‐fold and 1.7‐fold, respectively, in SCC9‐CisR cells compared with their parental cell lines, with significant differential alterations. A recent study reported significant alterations in the stemness markers CD24 and CD90 in lenvatinib‐resistant Hep‐3B and HuH‐7 cells with approximately 1.5‐2‐fold changes [32]. These data suggest that low‐fold changes in the mRNA levels for cancer stem cell markers significantly affect tumor drug resistance.

MCTs are proton‐linked membrane transport proteins that transport monocarboxylic acid molecules such as acetoacetate, pyruvate, and lactate in and out of cells [33]. Metabolic transformation, cancer aggressiveness, and chemoresistance are associated with MCT4 [34]. Furthermore, MCT4 overexpression facilitates tumor growth and offers a microenvironment that fosters tumor growth [35]. Additionally, MCT4 is a driver of oral squamous cell carcinoma [36]. Here, we discovered that MCT4 may be a target of HIF‐1α by analyzing DEGs in the GSE111585 database and the target of HIF‐1α with the iRegulon plugin. Moreover, MCT4 protein levels were overtly elevated in SCC9‐CisR cells, and Rha treatment, but not Cis treatment, decreased MCT4 protein levels in SCC9‐CisR cells. Nevertheless, Rha treatment did not affect MCT4 protein levels in SCC9‐CisR cells following HIF‐1α knockdown. A previous study revealed that HIF‐1α activates SLC16A3 (MCT4) by binding to an HRE [37]. Thus, we inferred that Rha mediates MCT4 protein levels by regulating HIF‐1α transcriptional activity.

The Wnt/β‐catenin is a signaling pathway located downstream of MCT4 [38]. Duan et al. have reported that MCT4 activates the WNT pathway to facilitate PD‐L1N‐glycosylation in MDA‐MB‐231 cells [38]. Hyperactivation of the Wnt/β‐catenin signaling pathway in cancer cells results in drug resistance and cancer recurrence in patients treated with conventional chemotherapy and radiotherapy [39]. Here, Rha plus Cis increased P‐GSK‐3β (Y216) and P‐β‐catenin protein levels in the cytoplasm and reduced β‐catenin protein levels in the nucleus compared with Cis alone, indicating that Rha plus Cis elevates GSK‐3β activity and represses nuclear translocation of β‐catenin. All results led us to conclude that Rha inhibits the Wnt/β‐catenin signaling by mediating the HIF‐1α/MCT4 axis, thus elevating the sensitivity of SCC9‐CisR cells to Cis. In the future, we plan to explore the effects of MCT4 depletion on Rha‐mediated changes in SCC9‐CisR cells. However, a limitation of the present study is that the key findings were derived from a single cisplatin‐resistant TSCC cell line. To strengthen the generalizability and translational relevance of our conclusions, future experiments will extend these investigations to additional TSCC models. Specifically, organ‐like models should be constructed with tumor cells isolated from patients with TSCC and transplanted into mice, followed by treatment of the mice with Rha in combination with Cis, which will yield results that have greater conviction and significance in the clinical setting. We are committed to establishing these advanced models to provide further evidence supporting the potential of Rha as a Cis adjuvant in TSCC therapy.

In conclusion, Rha plus Cis decreases cell viability, represses cell stemness, and promotes cell apoptosis in TSCC, thereby decreasing Cis resistance. Mechanistically, Rha represses the Wnt/β‐catenin signaling by mediating the HIF‐1α/MCT4 axis. This study suggests Rha as an effective adjuvant to Cis for TSCC treatment.

## Conflicts of Interest

The authors declare no conflicts of interest.

## Supporting information


**Figure S1.** Analysis of the role of HIF‐1α in Cis resistance in SCC9 cells.

**Table S1.** The primers for PCR reaction.

**Table S2.** The antibodies used in western blot.

## Data Availability

The data that support the findings of this study are available from the corresponding author upon reasonable request.
