# Supplementary material for: Rhaponitin Reverses Cisplatin Resistance and Impairs Cancer Stemness Through HIF‐1α/MCT4/Wnt Pathway in Tongue Squamous Cell Carcinoma
Source: Kaohsiung J Med Sci. 2025 Jul 3;41(11):e70069. doi: 10.1002/kjm2.70069 (PMC12622404; doi:10.1002/kjm2.70069)
Supplement: Supplementary file 1 — Figure S1. Analysis of the role of HIF‐1α in Cis resistance in SCC9 cells. [file KJM2-41-e70069-s002.docx]

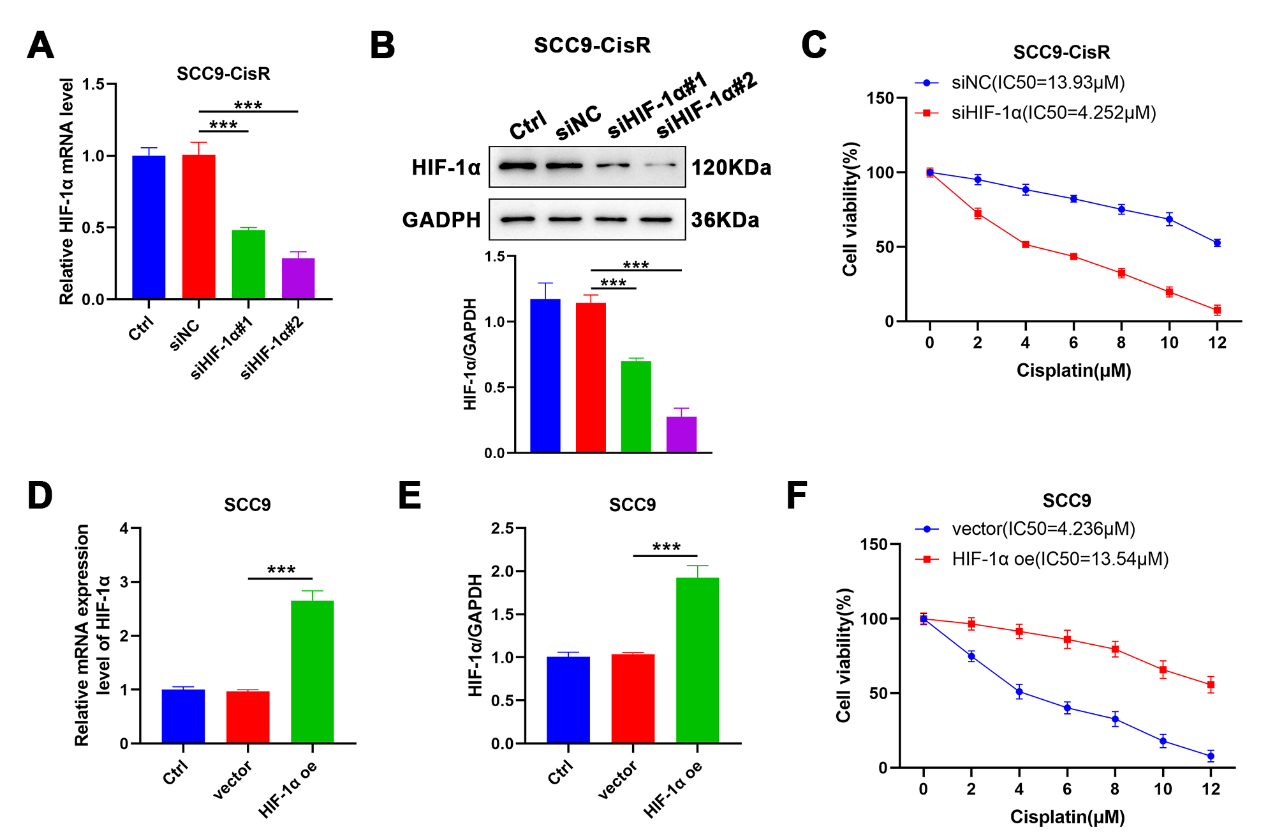


**Figure S1 Analysis of the role of HIF-1α in Cis resistance in** **SCC9 cells.** (A-B) The transcriptional and translational levels of HIF-1α in SCC9-CisR cells and SCC9-CisR cells transfected with siNC, siHIF-1α#1, or siHIF-1α#2 were detected by RT-qPCR and western blot (n = 3). (C) The IC_50_ values for Cis in SCC9-CisR cells transfected with siNC or siHIF-1α were analyzed by CCK-8 assays (n = 3). (D-E) After vector or HIF-1α oe introduction, HIF-1α mRNA and protein levels in SCC9 cells were assessed by RT-qPCR and western blot (n = 3). (F) Analysis of the IC_50_ values for Cis in SCC9 transfected with vector or HIF-1α oe by CCK-8 assays (n = 3). Data are presented as mean ± SEM. ^***^*p* < 0.001, one-way ANOVA (A, B, D, E); two-way ANOVA followed by Tukey’s post hoc test (C, F).
