# Supplementary material for: Rhaponitin Reverses Cisplatin Resistance and Impairs Cancer Stemness Through HIF‐1α/MCT4/Wnt Pathway in Tongue Squamous Cell Carcinoma
Source: Kaohsiung J Med Sci. 2025 Jul 3;41(11):e70069. doi: 10.1002/kjm2.70069 (PMC12622404; doi:10.1002/kjm2.70069)
Supplement: Supplementary file 2 — Table S1. The primers for PCR reaction. [file KJM2-41-e70069-s003.docx]

**Table S1 The primers for PCR reaction**

| Gene | Sequence |  |
| --- | --- | --- |
| CD44 | Forward | CCAGAAGGAACAGTGGTTTGGC |
|  | Reverse | ACTGTCCTCTGGGCTTGGTGTT |
| SOX2 | Forward | GCTACAGCATGATGCAGGACCA |
|  | Reverse | TCTGCGAGCTGGTCATGGAGTT |
| HIF-1α | Forward | GAACGTCGAAAAGAAAAGTCTCG |
|  | Reverse | CCTTATCAAGATGCGAACTCACA |
| GADPH | Forward | GGAGCGAGATCCCTCCAAAAT |
|  | Reverse | GGCTGTTGTCATACTTCTCATGG |
