# Supplementary material for: Rhaponitin Reverses Cisplatin Resistance and Impairs Cancer Stemness Through HIF‐1α/MCT4/Wnt Pathway in Tongue Squamous Cell Carcinoma
Source: Kaohsiung J Med Sci. 2025 Jul 3;41(11):e70069. doi: 10.1002/kjm2.70069 (PMC12622404; doi:10.1002/kjm2.70069)
Supplement: Supplementary file 3 — Table S2. The antibodies used in western blot. [file KJM2-41-e70069-s001.docx]

**Table S2 The antibodies used in western blot**

| Antibody | Manufacturer | Cat. No. |
| --- | --- | --- |
| HIF-1α | Beyotime | AG2135 |
| GADPH | Beyotime | AF0006 |
| MCT4 | Absin | abs124388 |
| P-GSK-3β | proteintech | 29125-1-AP |
| GSK-3β | Beyotime | AG751 |
| P-β-Catenin | Beyotime | AF5749 |
| β-Catenin | Beyotime | AF0069 |
| Lamin B1 | Beyotime | AF1408 |
| HRP-labeled Goat Anti-Mouse IgG | Beyotime | A0216 |
| HRP-labeled Goat Anti-Rabbit IgG | Beyotime | A0208 |
